# Supplementary material for: Effectiveness of interventions targeting air travellers for delaying local outbreaks of SARS-CoV-2
Source: J Travel Med. 2020 May 8;27(5):taaa068. doi: 10.1093/jtm/taaa068 (PMC7239177; doi:10.1093/jtm/taaa068)
Supplement: SI_taaa068 [file si_taaa068.docx]

*Title:* ***Effectiveness of*** ***interventions targeting air travellers for delaying local outbreaks of SARS-CoV-2 (Supporting Information)***

*Authors*: Samuel Clifford^1+^, Carl A.B. Pearson^1,2^, Petra Klepac^1^, Kevin Van Zandvoort^1^, Billy J. Quilty^1^, CMMID COVID-19 working group, Rosalind M. Eggo^1*^, Stefan Flasche^1*^

*Affiliations*: ^1^Centre for Mathematical Modelling of Infectious Diseases, Department of Infectious Disease Epidemiology, London School of Hygiene and Tropical Medicine, Keppel Street, WC1E 7HT London, UK
^2^South African DSI-NRF Centre of Excellence in Epidemiological Modelling and Analysis (SACEMA), University of Stellenbosch, 19 Jonkershoek Road, Stellenbosch, 7600, South Africa

*authors contributed equally
^+^corresponding author. [Sam.Clifford@lshtm.ac.uk](mailto:Sam.Clifford@lshtm.ac.uk)

### The following authors were part of the Centre for Mathematical Modelling of Infectious Disease 2019-nCoV working group. Each contributed in processing, cleaning and interpretation of data, interpreted findings, contributed to the manuscript, and approved the work for publication: Damien C Tully, Eleanor M Rees, Sam Abbott, Alicia Rosello, Thibaut Jombart, Graham Medley, Yang Liu, C Julian Villabona-Arenas, Kiesha Prem, Charlie Diamond, Stéphane Hué, Quentin J Leclerc, James D Munday, W John Edmunds, Akira Endo, Sophie R Meakin, Kathleen O'Reilly, David Simons, Katherine E. Atkins, Arminder K Deol, Emily S Nightingale, Adam J Kucharski, Mark Jit, Nikos I Bosse, Nicholas G. Davies, Joel Hellewell, Simon R Procter, Rachel Lowe, Amy Gimma, Anna M Foss, Rein M G J Houben, Fiona Yueqian Sun, Jon C Emery, Hamish P Gibbs, Timothy W Russell, Christopher I Jarvis, Gwenan M Knight, Sebastian Funk, Megan Auzenbergs.

Table S1 shows the percentage of the delays in Figures 1, S1-S3 that are infinitely long as a function of the traveller sensitisation. These values are independent of whether or not screening is performed, as well as $\lambda_{0}$and $k$.

Table S1: Percentage of delays which are infinitely long under varying levels of traveller sensitisation and values of the dispersion parameter,$k$. Results from $5\times10^{3}$ simulations.

|  | Effectiveness of Traveller Sensitisation | | |
| --- | --- | --- | --- |
| $k$ | 0% | 25% | 50% |
| 0.10 | 0.9% | 8.5% | 52.8% |
| 0.54 | 1.9% | 13.0% | 65.8% |
| 2.00 | 0.1% | 1.7% | 22.3% |

In Table S2 we present the full summary statistics of the sensitivity analysis.

Table S2. Sensitivity analysis summary statistics providing the inner 50% and 95% confidence intervals and medians (all rounded to the nearest day) for the estimated number of days an outbreak is delayed given an intervention consisting of a combination of traveller screening and sensitisation and contact tracing. Comparisons are made to no contact tracing and no screening (there are no “No screening” results at 0% sensitisation as this is the baseline against which comparisons are to be made).

| Dispersion parameter, *k* | Arrivals per week at time of intervention introduction, $\lambda_{0}$ | Effect of Traveller Sensitisation, $\varrho$ | Screening | Number of days for which the given percentage of delays are at least this long | | | | |
| --- | --- | --- | --- | --- | --- | --- | --- | --- |
|  |  |  |  | 97.5% | 75% | 50% | 25% | 2.5% |
| 0.1 | 0.1 | 0% | Exit and entry | <1 | 4 | 8 | 12 | 30 |
|  |  | 0% | Exit only | <1 | 4 | 7 | 11 | 28 |
|  |  | 25% | No screening | <1 | 1 | 3 | 6 | ∞ |
|  |  | 25% | Exit and entry | <1 | 8 | 13 | 19 | ∞ |
|  |  | 25% | Exit only | <1 | 7 | 12 | 18 | ∞ |
|  |  | 50% | No screening | <1 | 9 | ∞ | ∞ | ∞ |
|  |  | 50% | Exit and entry | 4 | 19 | ∞ | ∞ | ∞ |
|  |  | 50% | Exit only | 3 | 17 | ∞ | ∞ | ∞ |
|  | 1 | 0% | Exit and entry | <1 | 4 | 7 | 10 | 19 |
|  |  | 0% | Exit only | <1 | 3 | 6 | 9 | 19 |
|  |  | 25% | No screening | <1 | 1 | 3 | 5 | ∞ |
|  |  | 25% | Exit and entry | <1 | 7 | 11 | 16 | ∞ |
|  |  | 25% | Exit only | <1 | 6 | 10 | 14 | ∞ |
|  |  | 50% | No screening | <1 | 8 | ∞ | ∞ | ∞ |
|  |  | 50% | Exit and entry | 2 | 16 | ∞ | ∞ | ∞ |
|  |  | 50% | Exit only | 2 | 15 | ∞ | ∞ | ∞ |
|  | 10 | 0% | Exit and entry | <1 | 1 | 3 | 5 | 10 |
|  |  | 0% | Exit only | <1 | 1 | 2 | 4 | 9 |
|  |  | 25% | No screening | <1 | <1 | 1 | 2 | ∞ |
|  |  | 25% | Exit and entry | <1 | 2 | 5 | 8 | ∞ |
|  |  | 25% | Exit only | <1 | 2 | 4 | 8 | ∞ |
|  |  | 50% | No screening | <1 | 3 | ∞ | ∞ | ∞ |
|  |  | 50% | Exit and entry | <1 | 7 | ∞ | ∞ | ∞ |
|  |  | 50% | Exit only | <1 | 7 | ∞ | ∞ | ∞ |
|  | 100 | 0% | Exit and entry | <1 | <1 | <1 | 1 | 3 |
|  |  | 0% | Exit only | <1 | <1 | <1 | 1 | 2 |
|  |  | 25% | No screening | <1 | <1 | <1 | <1 | ∞ |
|  |  | 25% | Exit and entry | <1 | <1 | 1 | 2 | ∞ |
|  |  | 25% | Exit only | <1 | <1 | 1 | 2 | ∞ |
|  |  | 50% | No screening | <1 | <1 | ∞ | ∞ | ∞ |
|  |  | 50% | Exit and entry | <1 | 1 | ∞ | ∞ | ∞ |
|  |  | 50% | Exit only | <1 | 1 | ∞ | ∞ | ∞ |
| 0.54 | 0.1 | 0% | Exit and entry | <1 | <1 | 6 | 13 | 41 |
|  |  | 0% | Exit only | <1 | <1 | 5 | 12 | 41 |
|  |  | 25% | No screening | <1 | <1 | 1 | 7 | ∞ |
|  |  | 25% | Exit and entry | <1 | 4 | 12 | 22 | ∞ |
|  |  | 25% | Exit only | <1 | 3 | 10 | 20 | ∞ |
|  |  | 50% | No screening | <1 | 10 | ∞ | ∞ | ∞ |
|  |  | 50% | Exit and entry | <1 | 22 | ∞ | ∞ | ∞ |
|  |  | 50% | Exit only | <1 | 20 | ∞ | ∞ | ∞ |
|  | 1 | 0% | Exit and entry | <1 | <1 | 4 | 9 | 23 |
|  |  | 0% | Exit only | <1 | <1 | 3 | 8 | 21 |
|  |  | 25% | No screening | <1 | <1 | 1 | 4 | ∞ |
|  |  | 25% | Exit and entry | <1 | 3 | 8 | 14 | ∞ |
|  |  | 25% | Exit only | <1 | 2 | 7 | 13 | ∞ |
|  |  | 50% | No screening | <1 | 6 | ∞ | ∞ | ∞ |
|  |  | 50% | Exit and entry | <1 | 14 | ∞ | ∞ | ∞ |
|  |  | 50% | Exit only | <1 | 14 | ∞ | ∞ | ∞ |
|  | 10 | 0% | Exit and entry | <1 | <1 | 1 | 3 | 8 |
|  |  | 0% | Exit only | <1 | <1 | 1 | 2 | 7 |
|  |  | 25% | No screening | <1 | <1 | <1 | 1 | ∞ |
|  |  | 25% | Exit and entry | <1 | <1 | 2 | 5 | ∞ |
|  |  | 25% | Exit only | <1 | <1 | 2 | 4 | ∞ |
|  |  | 50% | No screening | <1 | 2 | ∞ | ∞ | ∞ |
|  |  | 50% | Exit and entry | <1 | 5 | ∞ | ∞ | ∞ |
|  |  | 50% | Exit only | <1 | 4 | ∞ | ∞ | ∞ |
|  | 100 | 0% | Exit and entry | <1 | <1 | <1 | <1 | 1 |
|  |  | 0% | Exit only | <1 | <1 | <1 | <1 | 1 |
|  |  | 25% | No screening | <1 | <1 | <1 | <1 | ∞ |
|  |  | 25% | Exit and entry | <1 | <1 | <1 | 1 | ∞ |
|  |  | 25% | Exit only | <1 | <1 | <1 | 1 | ∞ |
|  |  | 50% | No screening | <1 | <1 | ∞ | ∞ | ∞ |
|  |  | 50% | Exit and entry | <1 | 1 | ∞ | ∞ | ∞ |
|  |  | 50% | Exit only | <1 | 1 | ∞ | ∞ | ∞ |
| 2 | 0.1 | 0% | Exit and entry | <1 | <1 | 5 | 13 | 32 |
|  |  | 0% | Exit only | <1 | <1 | 4 | 12 | 31 |
|  |  | 25% | No screening | <1 | <1 | <1 | 4 | 28 |
|  |  | 25% | Exit and entry | <1 | 1 | 9 | 18 | 47 |
|  |  | 25% | Exit only | <1 | <1 | 8 | 17 | 46 |
|  |  | 50% | No screening | <1 | <1 | 12 | 40 | ∞ |
|  |  | 50% | Exit and entry | <1 | 10 | 23 | 53 | ∞ |
|  |  | 50% | Exit only | <1 | 9 | 21 | 53 | ∞ |
|  | 1 | 0% | Exit and entry | <1 | <1 | 3 | 7 | 17 |
|  |  | 0% | Exit only | <1 | <1 | 2 | 7 | 17 |
|  |  | 25% | No screening | <1 | <1 | <1 | 2 | 18 |
|  |  | 25% | Exit and entry | <1 | <1 | 5 | 11 | 28 |
|  |  | 25% | Exit only | <1 | <1 | 4 | 10 | 27 |
|  |  | 50% | No screening | <1 | <1 | 7 | 28 | ∞ |
|  |  | 50% | Exit and entry | <1 | 6 | 14 | 38 | ∞ |
|  |  | 50% | Exit only | <1 | 5 | 13 | 37 | ∞ |
|  | 10 | 0% | Exit and entry | <1 | <1 | <1 | 2 | 5 |
|  |  | 0% | Exit only | <1 | <1 | <1 | 1 | 5 |
|  |  | 25% | No screening | <1 | <1 | <1 | <1 | 7 |
|  |  | 25% | Exit and entry | <1 | <1 | 1 | 3 | 13 |
|  |  | 25% | Exit only | <1 | <1 | 1 | 3 | 13 |
|  |  | 50% | No screening | <1 | <1 | 1 | 12 | ∞ |
|  |  | 50% | Exit and entry | <1 | 1 | 4 | 18 | ∞ |
|  |  | 50% | Exit only | <1 | 1 | 4 | 18 | ∞ |
|  | 100 | 0% | Exit and entry | <1 | <1 | <1 | <1 | 1 |
|  |  | 0% | Exit only | <1 | <1 | <1 | <1 | 1 |
|  |  | 25% | No screening | <1 | <1 | <1 | <1 | 1 |
|  |  | 25% | Exit and entry | <1 | <1 | <1 | <1 | 3 |
|  |  | 25% | Exit only | <1 | <1 | <1 | <1 | 3 |
|  |  | 50% | No screening | <1 | <1 | <1 | 2 | ∞ |
|  |  | 50% | Exit and entry | <1 | <1 | 1 | 5 | ∞ |
|  |  | 50% | Exit only | <1 | <1 | <1 | 4 | ∞ |


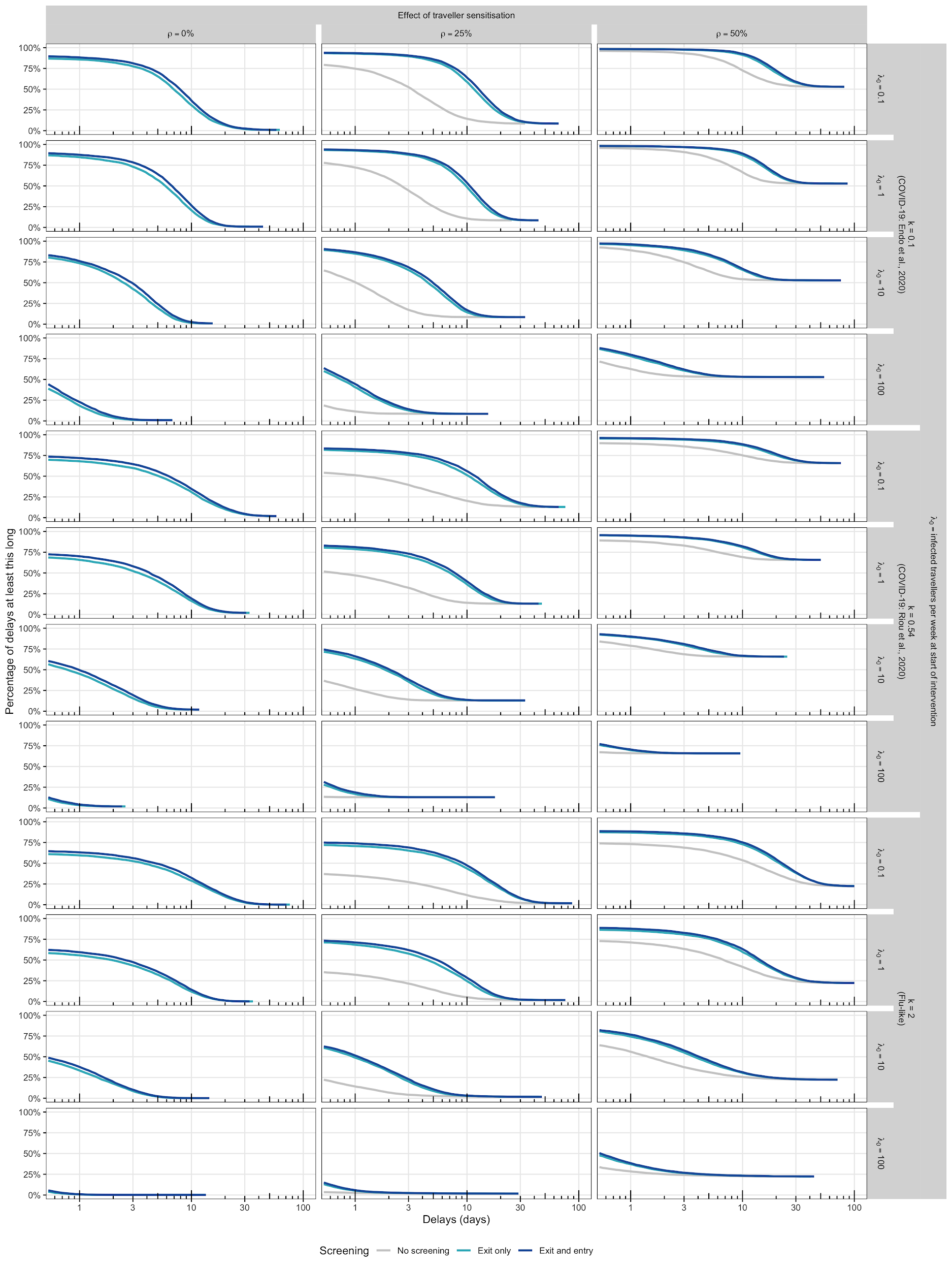


Figure S1: All scenarios for Figure 2 - complementary cumulative density functions (CCDF = 1-ECDF) of the estimated number of days an outbreak is delayed given an intervention consisting of a combination of traveller screening and sensitization and contact tracing. Within each panel, and for a given delay, the CCDF shows the percentage of simulations which result in a delay of at least that long for each screening regime (no screening, exit only, exit and entry). Comparisons are made to no contact tracing and no screening.


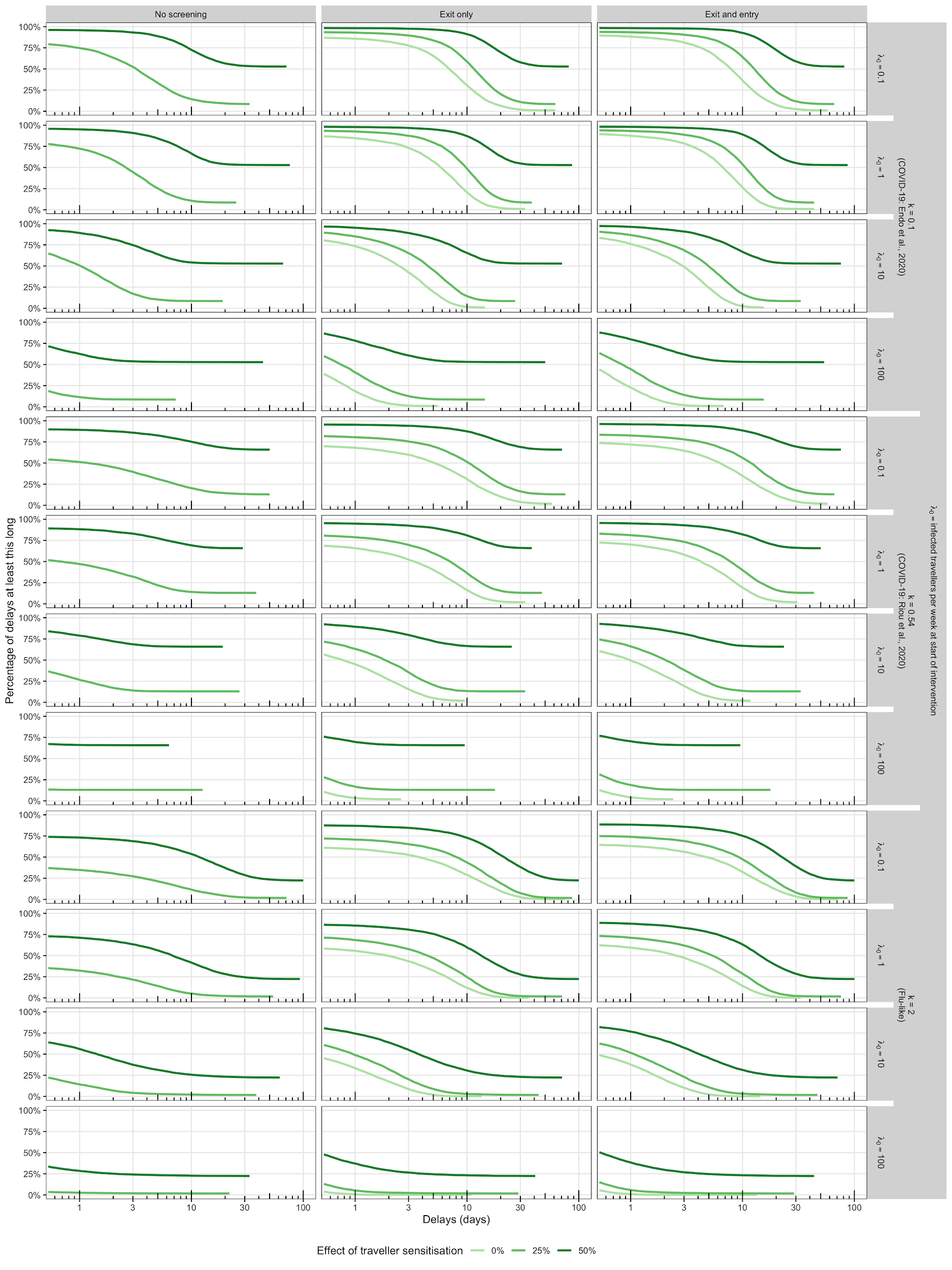


Figure S2: All scenarios for Figure 2 - complementary cumulative density functions (CCDF) of the estimated number of days an outbreak is delayed given an intervention consisting of a combination of traveller screening and sensitization and contact tracing. Within each panel, and for a given delay, the CCDF shows the percentage of simulations which result in a delay of at least that long for each level of traveller sensitisation (0%, 25%, 50%). Comparisons are made to no contact tracing and no screening.


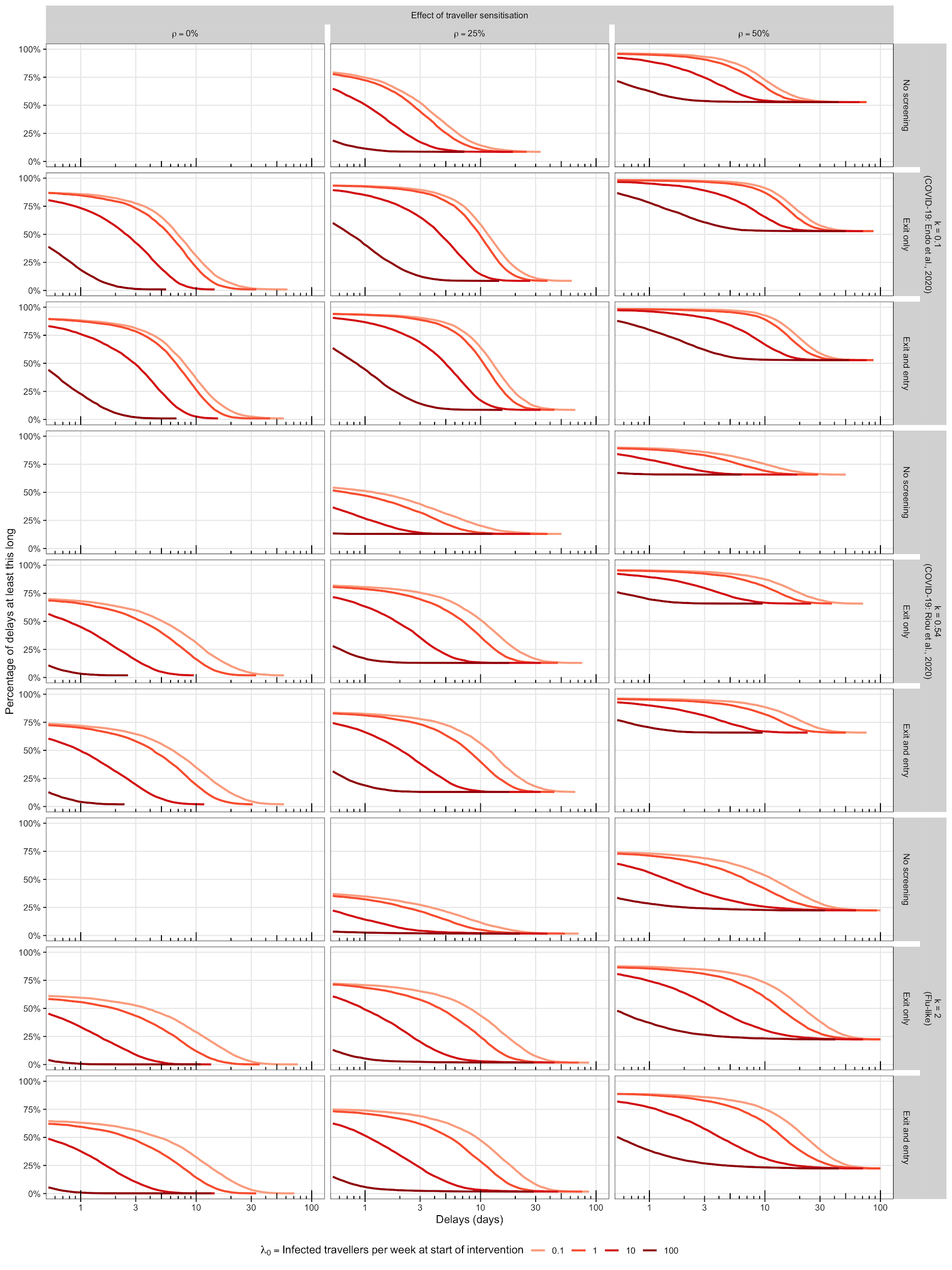


Figure S3: All scenarios for Figure 2 - complementary cumulative density functions (CCDF) of the estimated number of days an outbreak is delayed given an intervention consisting of a combination of traveller screening and sensitization and contact tracing. Within each panel, and for a given delay, the CCDF shows the percentage of simulations which result in a delay of at least that long for each rate of arrival of infected travellers at the time interventions are introduced (0.1, 1, 10 and 100 per week). Comparisons are made to no contact tracing and no screening.


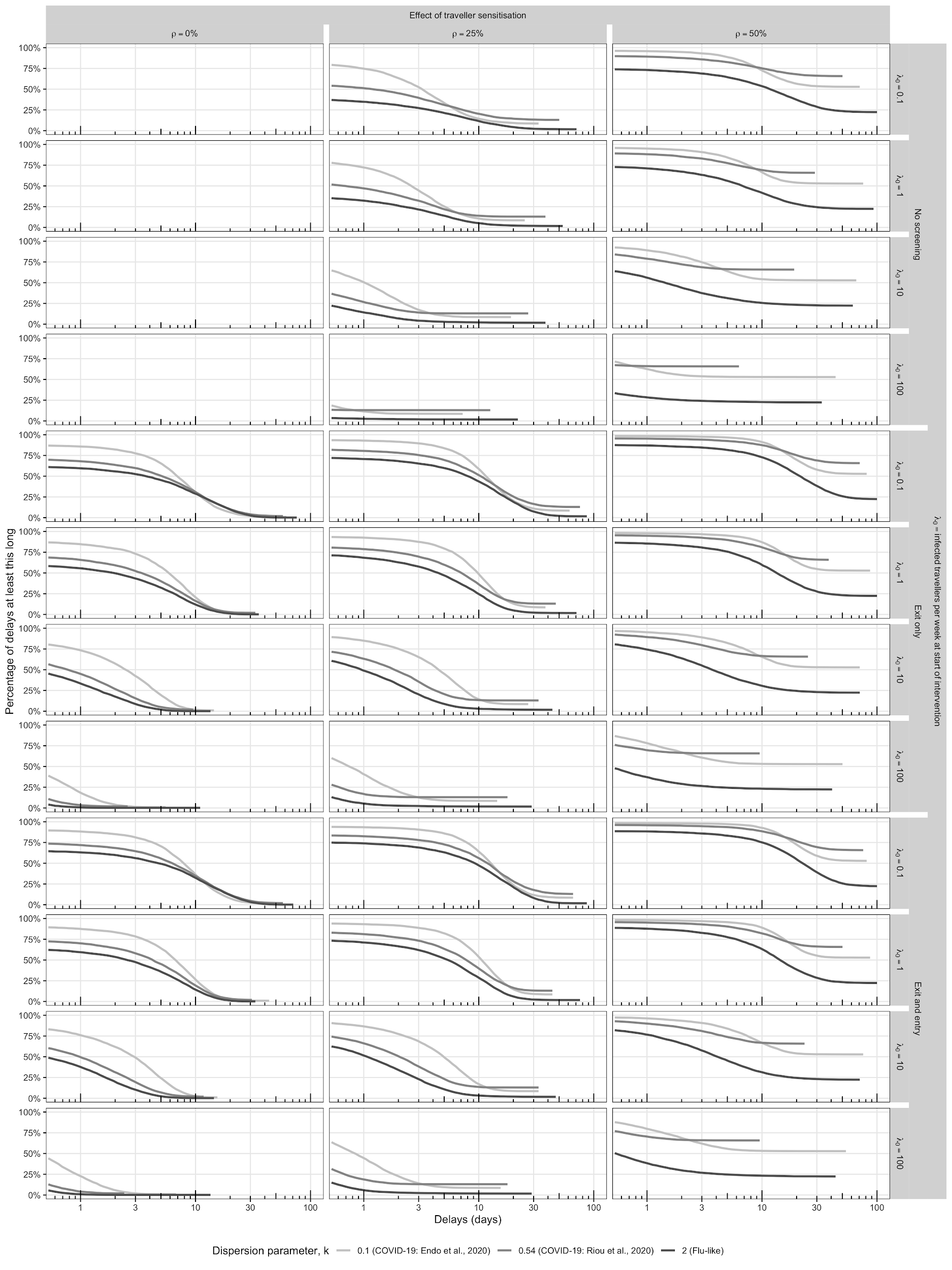


Figure S4: All scenarios for Figure 2 - complementary cumulative density functions (CCDF) of the estimated number of days an outbreak is delayed given an intervention consisting of a combination of traveller screening and sensitization and contact tracing. Within each panel, and for a given delay, the CCDF shows the percentage of simulations which result in a delay of at least that long for each dispersion parameter considered. Comparisons are made to no contact tracing and no screening.
